# Accuracy of the Interpolated Surface Mesh Method for OEP Volume Measurement

**DOI:** 10.3390/s26185709

**Published:** 2026-09-09

**Authors:** Laurent Gaillard, Laurent Stubbe, Damien Riquet, Nicolas Houel

**Affiliations:** 1 PSMS, UR 7507, Université de Reims Champagne-Ardenne, 51097 Reims, France; laurent.gaillard1@etudiant.univ-reims.fr (L.G.); damien.riquet@etudiant.univ-reims.fr (D.R.); nicolas.houel@univ-reims.fr (N.H.); 2 ESO-Paris Recherche, Ecole Supérieure d’Ostéopathie-Paris, 77420 Champs-sur-Marne, France; 3 CIAMS, Université Paris-Saclay, 91405 Orsay, France; 4 URCATech-MoCAP, USAR4476, Université de Reims Champagne-Ardenne, 51097 Reims, France

**Keywords:** optoelectronic plethysmography, breathing kinematics, chest wall modeling, metrology

## Abstract

**Highlights:**

The proposed interpolation method enhances surface resolution for breathing kinematic analysis by capturing local chest wall deformations. It can be integrated into the current OEP workflow without compromising the accuracy of volume measurements.

**What are the main findings?**

- The interpolation method is as accurate as the conventional method for small and large volume variations.
- The systematic bias introduced by OEP volume measurement was reduced by the interpolation method for larger volume variations.

**What are the implications of the main findings?**

- The update of the current OEP algorithm with the recent interpolation method is not expected to affect the volume measurement accuracy.
- The presented interpolation method does not require the use of third-party software, thus facilitating its implementation in the current workflow.

**Abstract:**

*Background*: Optoelectronic plethysmography (OEP) is a non-invasive technique that can be used to assess both respiratory volumes and chest wall kinematics. An interpolation surface method (ISM) has been recently developed to enhance OEP kinematic analysis. The aim of this study was to test whether ISM is as accurate as the conventional method (CM) for volume measurement when compared with conventional spirometry. *Methods*: Pneumotachographs and OEP were recorded simultaneously on a healthy subject who performed quiet and deep breathing. *Results*: In total, 124 tidal volumes (TV) and 50 vital capacities (VC) were obtained. ISM introduced a lower bias for both TV and VC, with narrower limits of agreement for CM in both conditions. Mean discrepancies were systematically <2.15%. Correlation coefficients were in the interval [0.978; 0.991], with an ICC in the interval [0.968; 0.991] for both TV and VC. *Conclusions*: ISM and CM have similar volume measurement accuracies. ISM preserves the accuracy of OEP volume measurements and its software environment. Although ISM represents a promising approach for breathing motion analysis, its contribution to improving kinematic analysis remains to be evaluated across a broader range of morphologies and breathing patterns.

## 1. Introduction

Optoelectronic plethysmography (OEP) captures the 3D motion of points located at the surface of the chest wall and converts it into respiratory volume measurements and chest wall kinematic analyses [1]. OEP overcomes some of the main limitations and biases attributable to conventional spirometry, considered a gold-standard technique in pulmonary function testing [2,3,4]. Indeed, OEP, being non-invasive, can be performed in various positions and experimental conditions [1], without tubes, mouthpieces or nose clips [2]. The patient is free to breathe in the most convenient way [5], and OEP is also suitable for non-cooperative patients such as, for instance, newborns [6] or intensive care unit patients [7]. The motion capture system avoids signal drift due to numerical integrators, affecting pneumotachographs, or measurement errors due to variations in air temperature, pressure and humidity, affecting volume displacement spirometers [7,8]. The breathing volume measurement made with OEP relies on the fundamental assumption that variations in chest wall volume give rise to a pressure gradient between the atmosphere and the alveoli, at the onset of airflow production, in accordance with Boyle’s law [9]. A number of validation studies have pointed out a high level of agreement between OEP and spirometry, thereby assessing the accuracy of OEP for volume measurement [1,2,3]. The comparison between conventional method (CM) OEP and spirometry resulted in maximum differences of <4% for all studies, with high correlation between the two techniques [1].

OEP kinematic analysis provides genuine and unique supplementary materials of high interest for diagnostic and therapeutic purposes that are not as attainable using other techniques [10]. The freedom in choosing a geometric model to analyze chest wall motion has led to the compartmentalization of the trunk in order to analyze the contribution of each region to the total chest wall motion [5]. The three-compartment model, composed of the pulmonary rib cage, the abdominal rib cage and abdomen, has been extensively used to detect and measure paradoxical movements [11,12,13] and to test the effect of exercise and body positions on breathing kinematics [14,15,16]. The six-compartment model, composed of the left and right parts of the previous three, addresses the symmetry of breathing motion [10,17]. Nevertheless, OEP kinematic analysis suffers from a large surface resolution, which is inappropriate for detecting local variations and asynchronies [18]. We recently proposed a new computational method based on surface interpolation (ISM) that increases the surface resolution, with an adjustable number of points and element sizes. This technique can be easily included in the current OEP workflow and requires fewer markers than the conventional method. This method is intended to shift the focus of the OEP kinematic analysis from a regional motion converted into volume variations to a local analysis of each moving point located at the surface of the chest wall. However, the enhancement of kinematic analysis with the ISM must not be achieved at the expense of the accuracy of volume measurements. ISM has to perform at least as well as the conventional OEP method in terms of volume measurement in order to be approved.

The aim of this study is to compare the volume measurement accuracy of ISM and CM against conventional spirometry.

## 2. Materials and Methods

### 2.1. Optoelectronic Plethysmography

Eighty-nine passive markers were fixed with double-sided adhesive to a patient’s trunk [1]. The markers are positioned at the intersection of seven horizontal rows and twelve vertical columns. The rows are at the level of the clavicular notch, the manubriosternal joint, the nipples, the xiphoid process, the lower costal margin, the umbilicus and the anterior iliac spine process. The vertical columns are the anterior and posterior midlines, the anterior and posterior axillary lines on both sides, the four equidistant lines from the midlines and the previous axillary lines, and finally the two midaxillary lines [19]. The two midaxillary lines are composed of the five lowest horizontal lines given the presence of the scapulohumeral complex. Extra markers are placed on the clavicular lines, the pectoral region, the scapular region and the epigastric region to increase the marker density where the markers are well separated [19]. As the patient breathes in and out, the markers move, and the optoelectronic system captures the markers’ motion by recording their 3D coordinates over time.

**Figure 1:**
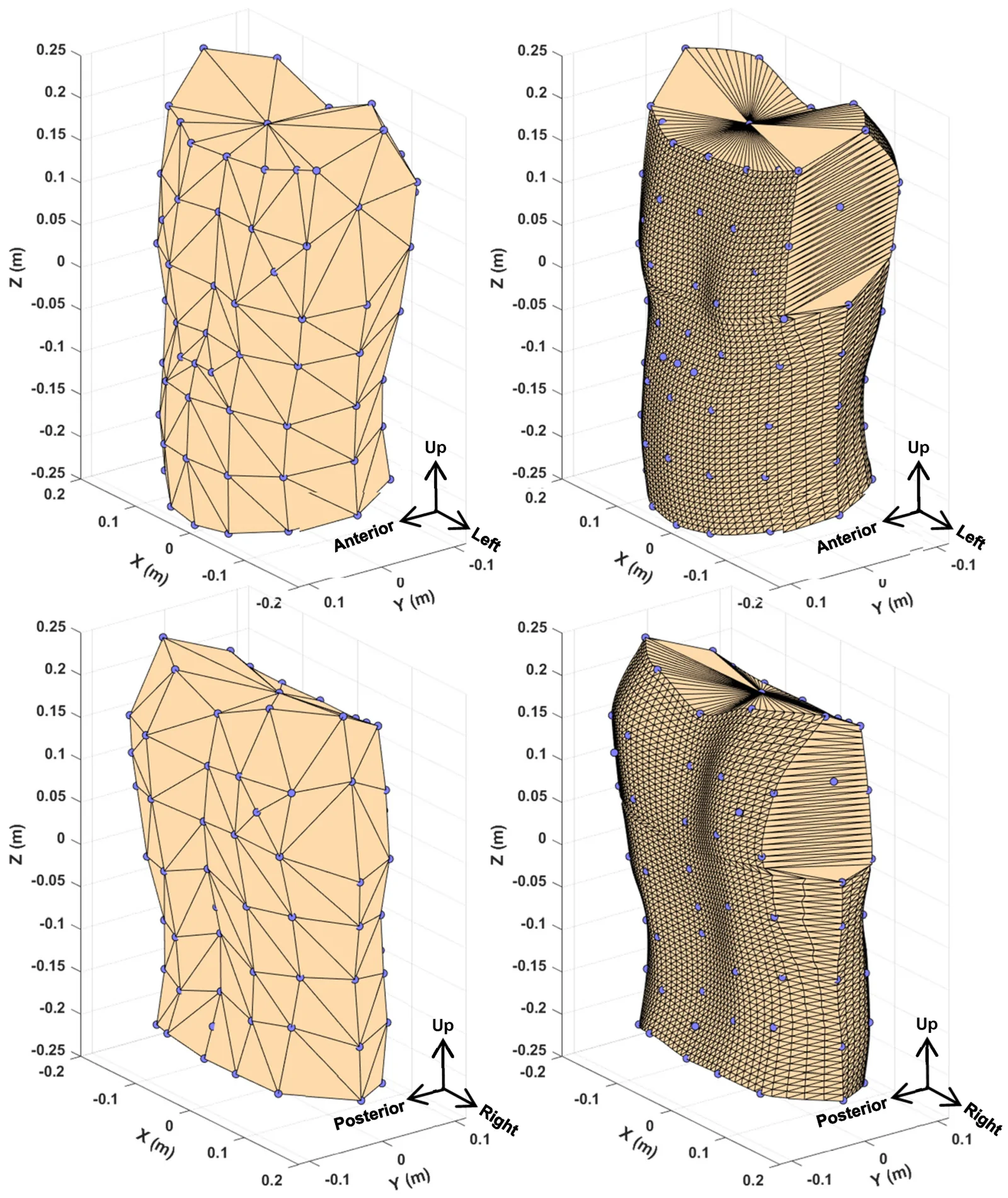
Surface mesh in CM (**left panels**) and in ISM (**right panels**). Blue dots correspond to 89 markers attached to the participant’s skin and the four virtual points used in CM to close the chest wall surface. (**Upper**) panels correspond to an anterior left-side view. (**Lower**) panels correspond to a posterior right-side view.

The CM converts the displacement of the markers into a volume measurement by applying the Gauss theorem [7]. The surface delineated by the markers is triangulated. Virtual points are necessarily added at the location of the shoulders, the head and the pelvic floor to close the surface [19]. The Gauss theorem then computes the volume enclosed by this closed-triangulated surface. The surface mesh in CM is composed of 93 vertices and 182 triangles (Figure 1).

The ISM takes advantage of the vertical and horizontal alignments of the original markerset to perform interpolation using the modified Akima method [20]. Before interpolation, a virtual central column composed of seven centers is added. Each center is the average point of every ellipsoid horizontal row [21]. Two interpolations are performed, first vertically and then horizontally. A slice of the trunk is composed of the collection of points from two adjacent horizontal rows and their respective centroid. These slices are decomposed into prisms with the two centroids corresponding to the vertices and the four adjoining points on the thoraco-abdominal surface corresponding to their base. The volume of each prism was calculated by adding together the volumes of its three constituent tetrahedra, using the Lagrange formula [21]. The total chest wall volume is equal to the sum of all prisms’ volumes, also known as the Prism-Based Method (PBM) [21]. The interpolation method allows us to choose the final number of points, thereby adjusting the surface resolution. For this study, a 60-point vertical interpolation and a 40-point horizontal interpolation resulted in a surface mesh composed of 4694 vertices. The number of points was chosen to set the surface resolution to around 1 cm^2^ per element, and the height of each slice was around 1 cm. The total chest wall volume was decomposed into 4557 prisms (Figure 1).

As spirometry measures airflow at the mouth, the single-compartment model was considered the most appropriate for comparing both OEP methods to spirometry.

### 2.2. Experimental Protocol

**Figure 2:**
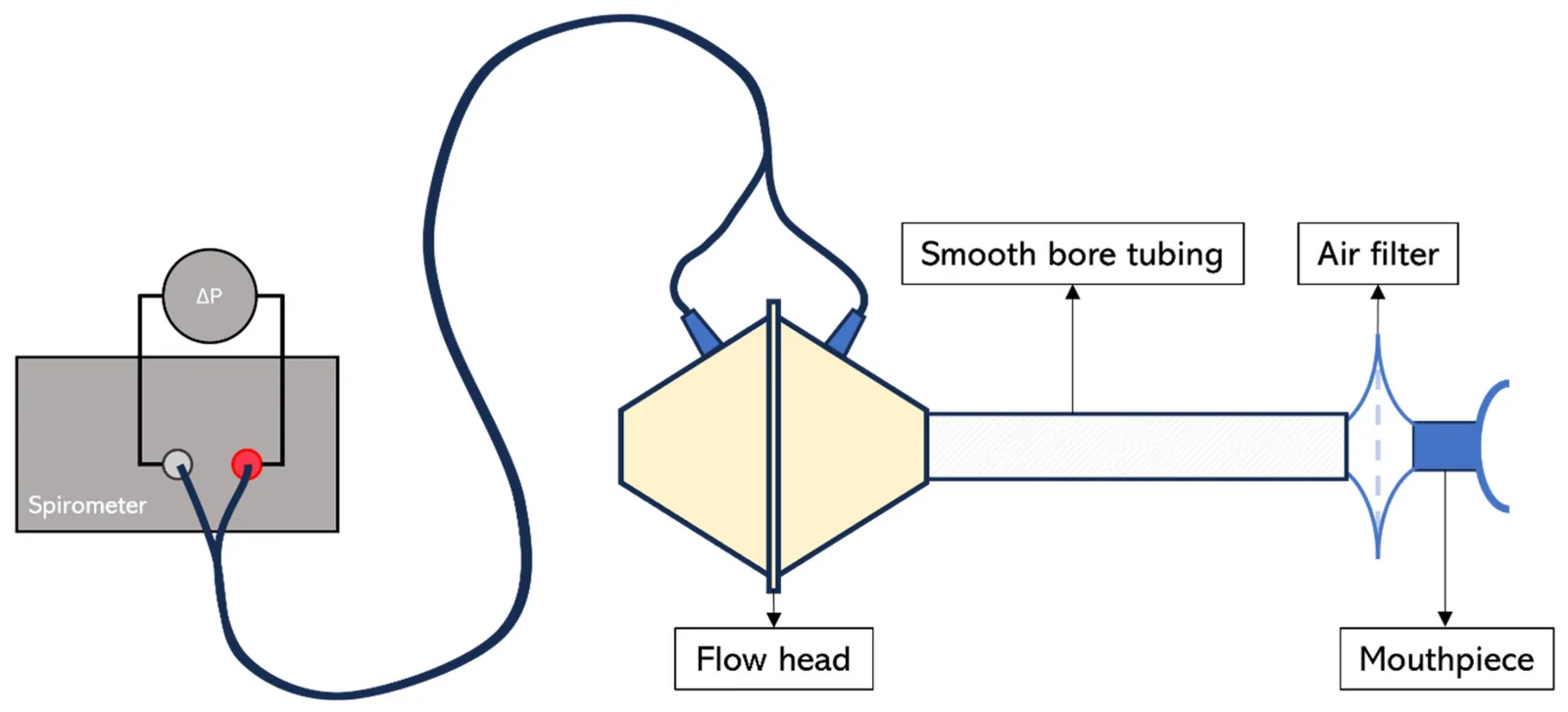
Spirometer FE141 configuration. The air filter captures droplets, condensation and macroscopic dust.

The optoelectronic system comprised eight cameras (Optitrack Flex13, Corvallis, OR, USA) positioned at 2–3 m from the center of the reconstructed field, 2 m high, with three cameras in front of the subject, three behind and two on the sides. The sampling frequency was 30 Hz. The system calibration was performed one hour after switching on the system, according to the most recent recommendation [22]. The experimental error of the optoelectronic system was 0.078 mm, which is consistent with previous experiments performed in our laboratory [23,24]. The FE141 spirometer (Labchart, ADInstruments, Dunedin, New Zealand) is a differential pressure amplifier that measures respiratory flow rates using a pneumotach MLT 1000 flow head (Figure 2). The spirometer was calibrated with a 3 L syringe before each measurement. The pressure resolution of this spirometer varies from 3.11 × 10^−4^ to 7.8 × 10^−3^ Pa for pressure ranges from 9.95 to 248.84 Pa. The sampling frequency was 1000 Hz. A low-pass fourth-order Bessel filter with a 10 Hz cut-off was applied.

**Figure 3:**
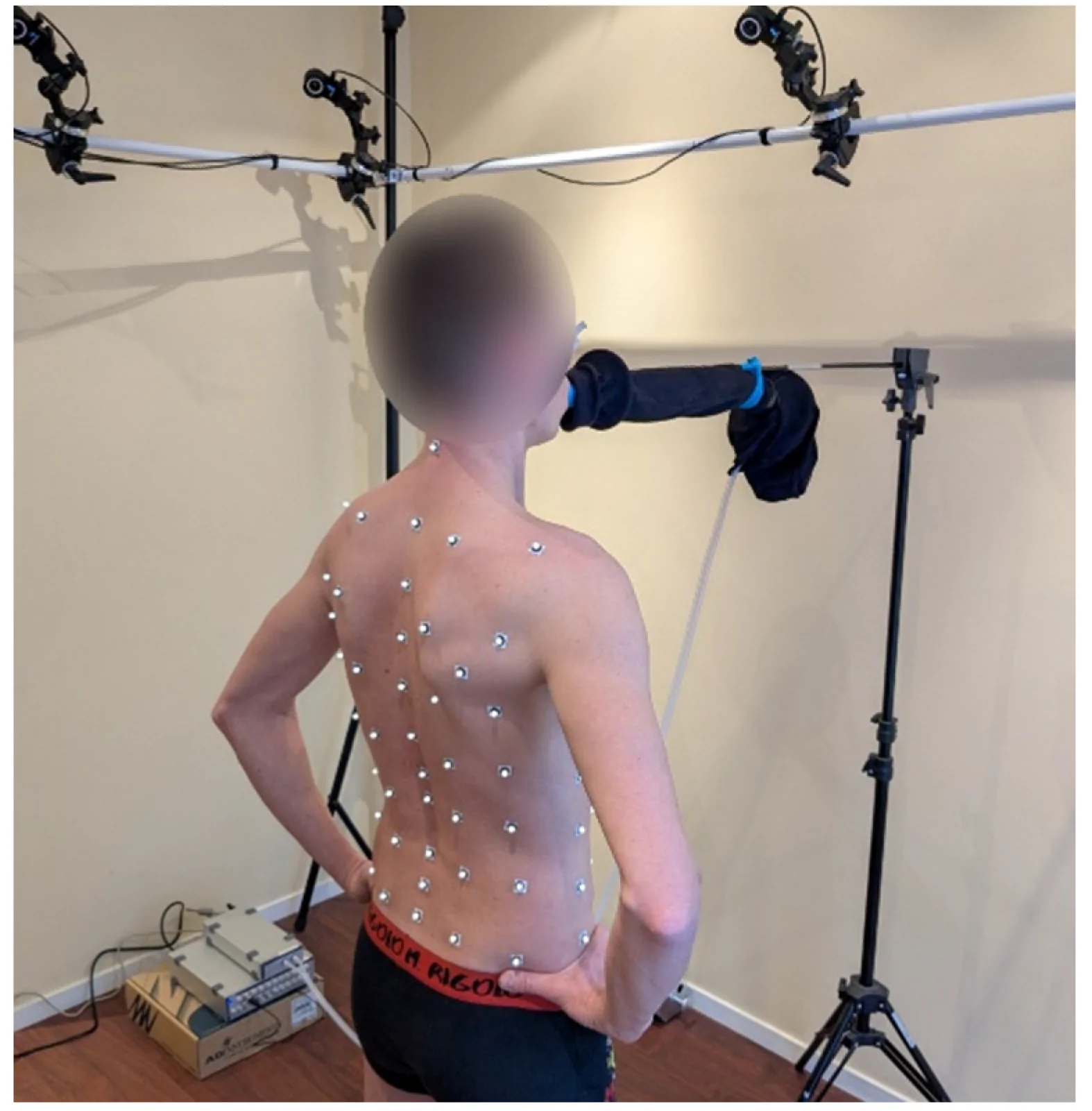
Experimental configuration. The subject is standing in a comfortable stance, breathing through the mouthpiece while the optoelectronic system records markers’ 3D coordinates.

A healthy volunteer was recruited in the laboratory. This pilot study was conducted in accordance with the ethical principles outlined in the Declaration of Helsinki and its subsequent amendments. The patient’s written informed consent was obtained. The subject was standing at the center of the reconstructed volume. He wore a nose clip and breathed through a mouthpiece, applying pressure with their lips to ensure a seal. The subject was invited to find a comfortable stance, after which the mouthpiece was positioned in front of him to minimize the constraints produced by the spirometer measurement on his posture and breathing motion (Figure 3).

The subject performed ten repetitions of quiet breathing for one minute, with 1–2 min of rest in between. Then, the subject performed five repetitions of ten slow vital capacities with 1–2 min of rest in between. We chose to use slow vital capacities to not only minimize blood shift [25] and dynamic airway compression [26] that might occur during forced expiration but also to avoid non-linearities affecting pneumotachographs at high flow rates [27]. The subject was asked to inhale and exhale into the mouthpiece with a slow flow rate that was as constant as possible. Control of the amplitude and frequency of both breathing exercises was left to the subject’s discretion. The spirometer was calibrated before each exercise during the subject’s resting time. A maximal exhalation at the beginning of each measurement was used to synchronize the spirometer and the optoelectronic system.

### 2.3. Data Analysis

The markers’ trajectories were labeled with motion capture software (Motive v 2.3, Optitrack, Corvallis, OR, USA) and exported as a .c3d file. The spirometer volume measurement signals were exported from the Labchart software (LabChart Pro 8, Labchart, ADInstruments, Dunedin, New Zealand) as a .mat file. The .c3d and .mat files were then imported into MATLAB 2024b. The OEP volume computations were performed using CM and ISM. The spirometer files were downsampled with a polyphase antialiasing filter to reach a sampling frequency of 30 Hz. The three signals were synchronized with the full expiration performed at the beginning of each measurement. Cross-correlation was used to control the synchronization and ensure that there was no lag between the signals. The tidal volumes (TV) and vital capacities (VC) were computed as the difference between end-inspiratory and end-expiratory volumes for each respiratory cycle.

The usual kinematic analysis performed in OEP quantifies the motion of a defined number of compartments, generally 3 based on the mechanics of breathing [28], expressed in liters or in percentages corresponding to the contribution of each compartment to the total chest wall motion [1]. The increased number of points provided by ISM was used to focus on the displacement of each point delineating the chest wall surface in order to characterize the local deformation of the chest wall. A shift in the reference frame was applied from world reference to the barycenter of the model. Then, the Cartesian coordinates of all points, for both CM and ISM, were converted into spherical coordinates. For vital capacities, the amplitude of the radial displacement of each point (in m) was represented with a colormap.

### 2.4. Statistical Analysis

The three methods of measuring TV and VC (Spiro, CM and ISM) were compared in pairs. The direction and the strength of the relationship between the different methods were reported in accordance with the Guidelines for Reporting Reliability and Agreement Studies [29]. Linear regressions and Pearson’s correlation coefficients with 95% CIs were computed. Agreement between methods was assessed using a Bland–Altman (B&A) analysis [30], completed using an intra-class correlation coefficient (ICC) type A-1 [31] (two-way mixed effects model, absolute agreement, single measures). The mean percentage discrepancy between OEP methods and the reference volume was calculated as in reference [7]: D(%) = [(ΔVSpiro − ΔVOEP,X)/ΔVSpiro] × 100, with X standing for CM or ISM. The results are presented as mean ± SD. Paired-samples t-tests were performed to assess whether the mean difference between methods differed significantly from zero. For all analyses, statistical significance was set at *p* < 0.01.

## 3. Results

One volunteer participated in this study. The participant was a 36-year-old male, 1.75 m, 58 kg. No history concerning the lungs, the respiratory tract, the heart, the viscera or the musculoskeletal structures composing the trunk was reported.

**Figure 4:**
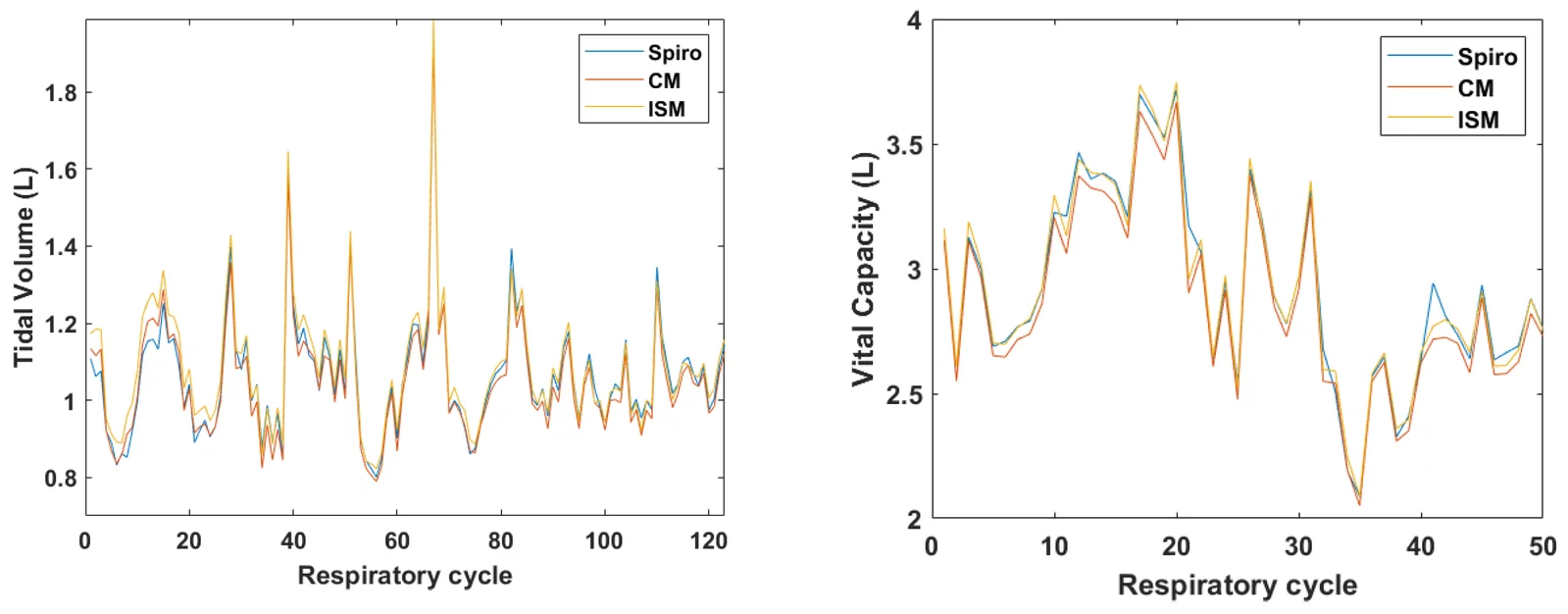
Tidal volumes (**left**) and vital capacities (**right**) measured with the three methods.

The participant performed 124 respiratory cycles during quiet breathing and 50 respiratory cycles during deep breathing (Figure 4). The respiratory volumes were TV_Spiro_ = 1.062 ± 0.155 L, TV_OEP,CM_ = 1.046 ± 0.154 L, TV_OEP,ISM_ = 1.084 ± 0.159 L; VC_Spiro_ = 2.922 ± 0.379 L, VC_OEP,CM_ = 2.865 ± 0.370 L, and VC_OEP,ISM_ = 2.921 ± 0.382 L.

**Figure 5:**
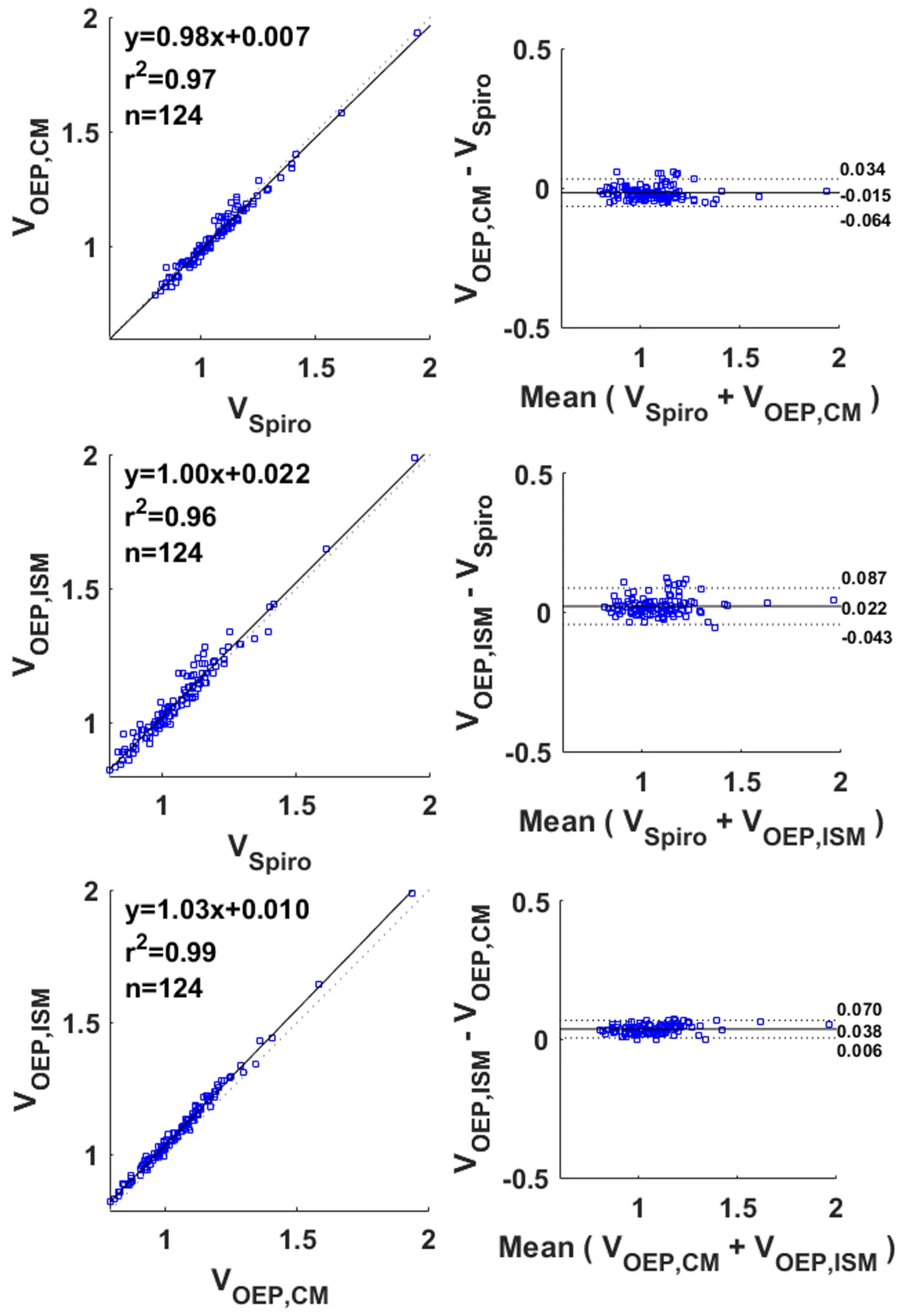
Linear regression (**left**) and Bland–Altman analysis (**right**) for tidal volumes. The (**upper**) panels correspond to CM vs. spirometry; the (**middle**) panels are ISM vs. spirometry; the (**lowest**) are ISM vs. CM.

For TV, the linear regression equations were V_OEP,CM_ = 0.98 × V_Spiro_ + 0.007 (r^2^ = 0.97), V_OEP,ISM_ = 1.00 × V_Spiro_ + 0.022 (r^2^ = 0.96) and V_OEP,ISM_ = 1.03 × V_OEP,CM_ + 0.010 (r^2^ = 0.99) (Figure 5). The correlation coefficients were 0.987 ([0.981; 0.991], *p* < 0.001) for CM vs. Spiro, 0.978 ([0.969; 0.984], *p* < 0.001) for ISM vs. Spiro, and 0.995 ([0.993; 0.996], *p* < 0.001). The B&A analysis of CM vs. Spiro showed a significant systematic bias of −0.015 L (*p* < 0.001, 95%CI = [−0.019;−0.011] L) with limits of agreement (LOA) of [−0.064; 0.034] L (Figure 5) and ICC = 0.982 (*p* < 0.001). The B&A analysis of ISM vs. Spiro showed a significant systematic bias of +0.022 L (*p* < 0.001, 95%CI = [0.016; 0.028] L) with LOA of [−0.043; 0.087] L (Figure 5) and ICC = 0.968 (*p* < 0.001). The B&A analysis of ISM vs. CM showed a significant systematic bias of +0.038 L (*p* < 0.001, 95%CI = [0.035; 0.041] L) with LOA of [0.006; 0.070] L (Figure 5) and ICC = 0.966 (*p* < 0.001). The discrepancies for TV measurements were +1.44 ± 2.35% for CM vs. Spiro, −2.15 ± 3.14% for ISM vs. Spiro, and +3.63 ± 1.48% for ISM vs. CM.

**Figure 6:**
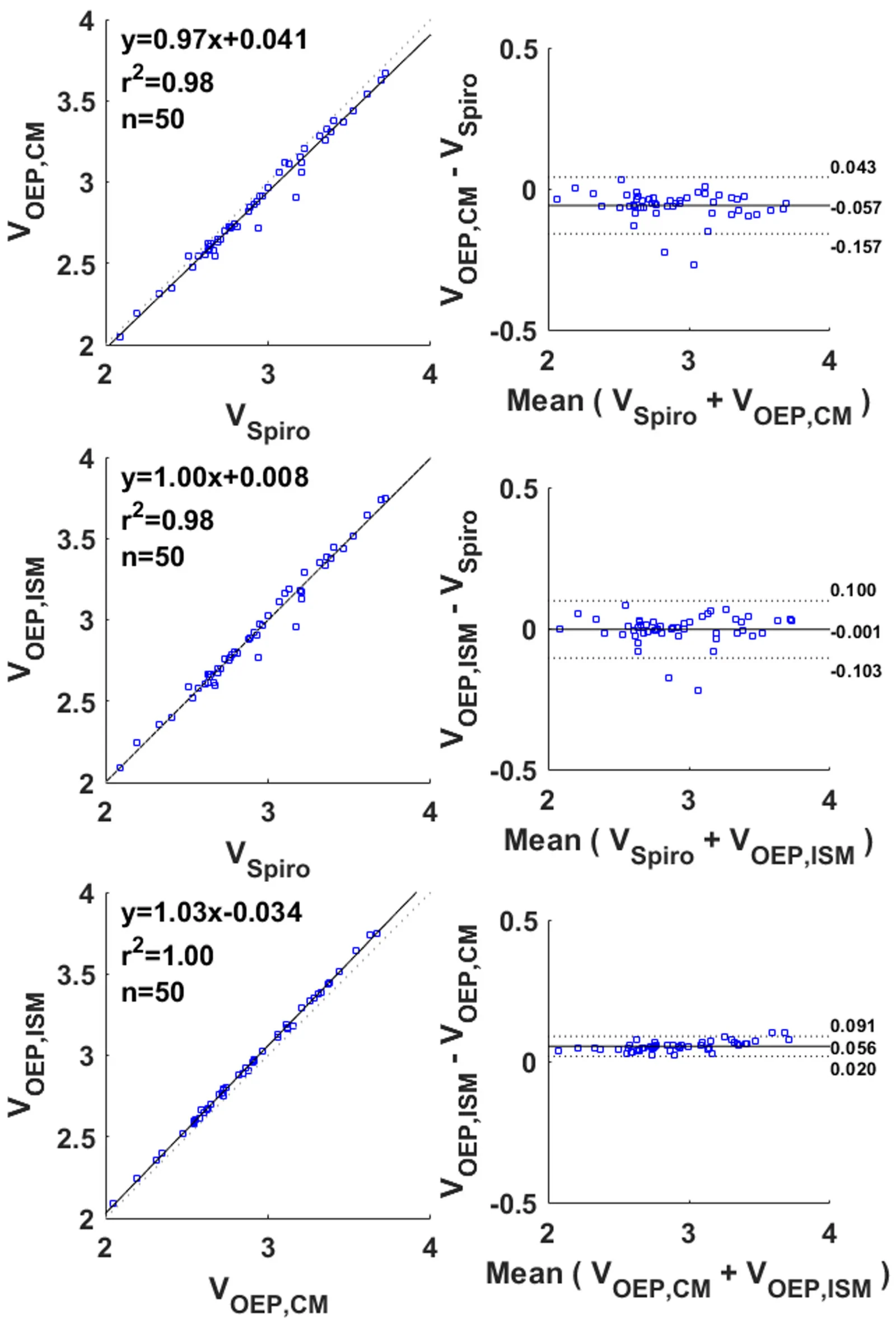
Linear regression (**left**) and Bland–Altman analysis (**right**) for vital capacities. The (**upper**) panels correspond to CM vs. spirometry; the (**middle**) panels are ISM vs. spirometry; the (**lowest**) are ISM vs. CM.

For VC, the linear regression equations were V_OEP,CM_ = 0.97 × V_Spiro_ + 0.041 (r^2^ = 0.98), V_OEP,ISM_ = 1.00 × V_Spiro_ + 0.008 (r^2^ = 0.98), and V_OEP,ISM_ = 1.03 × V_OEP,CM_ + 0.034 (r^2^ = 1.00) (Figure 6). The correlation coefficients were 0.991 ([0.984; 0.995], *p* < 0.001) for CM vs. Spiro, 0.991 ([0.984; 0.995], *p* < 0.001) for ISM vs. Spiro, and 0.999 ([0.998; 1.000], *p* < 0.001) for ISM vs. CM. The B&A analysis of CM vs. Spiro showed a significant systematic bias of −0.057 L (*p* < 0.001, 95%CI = [−0.071;−0.042] L), with an LOA of [−0.157; 0.043] L (Figure 6) and an ICC = 0.980 (<0.001). The B&A analysis of ISM vs. Spiro showed a non-significant systematic bias of −0.001 L (*p* = 0.86, 95%CI = [−0.016; 0.013] L) with LOA of [−0.103; 0.100] L (Figure 6) and ICC = 0.991 (*p* < 0.001). The B&A analysis of ISM vs. CM showed a significant systematic bias of +0.056 L (*p* < 0.001, 95%CI = [0.050; 0.061] L) with LOA of [−0.020; 0.091] L (Figure 6) and ICC = 0.988 (*p* < 0.001). The discrepancies for VC measurements were +1.91 ± 1.67% for CM vs. Spiro, +0.03 ± 1.74% for ISM vs. Spiro, and +1.92 ± 0.49% for ISM vs. CM.

The outcomes assessing the respective agreement of CM and ISM with spirometry, for both TV and VC, are summarized in Table 1.

The comparison between the CM and ISM resulted in a systematic overestimation of ISM for both absolute volumes and volume variations. For absolute volumes, over the 37,453 frames recorded, the mean ISM overestimation was +1.79 ± 0.22%. Concerning the volume variations, the overestimation of ISM was +3.63 ± 1.48% for TV, and +1.92 ± 0.49% for VC.

**Figure 7:**
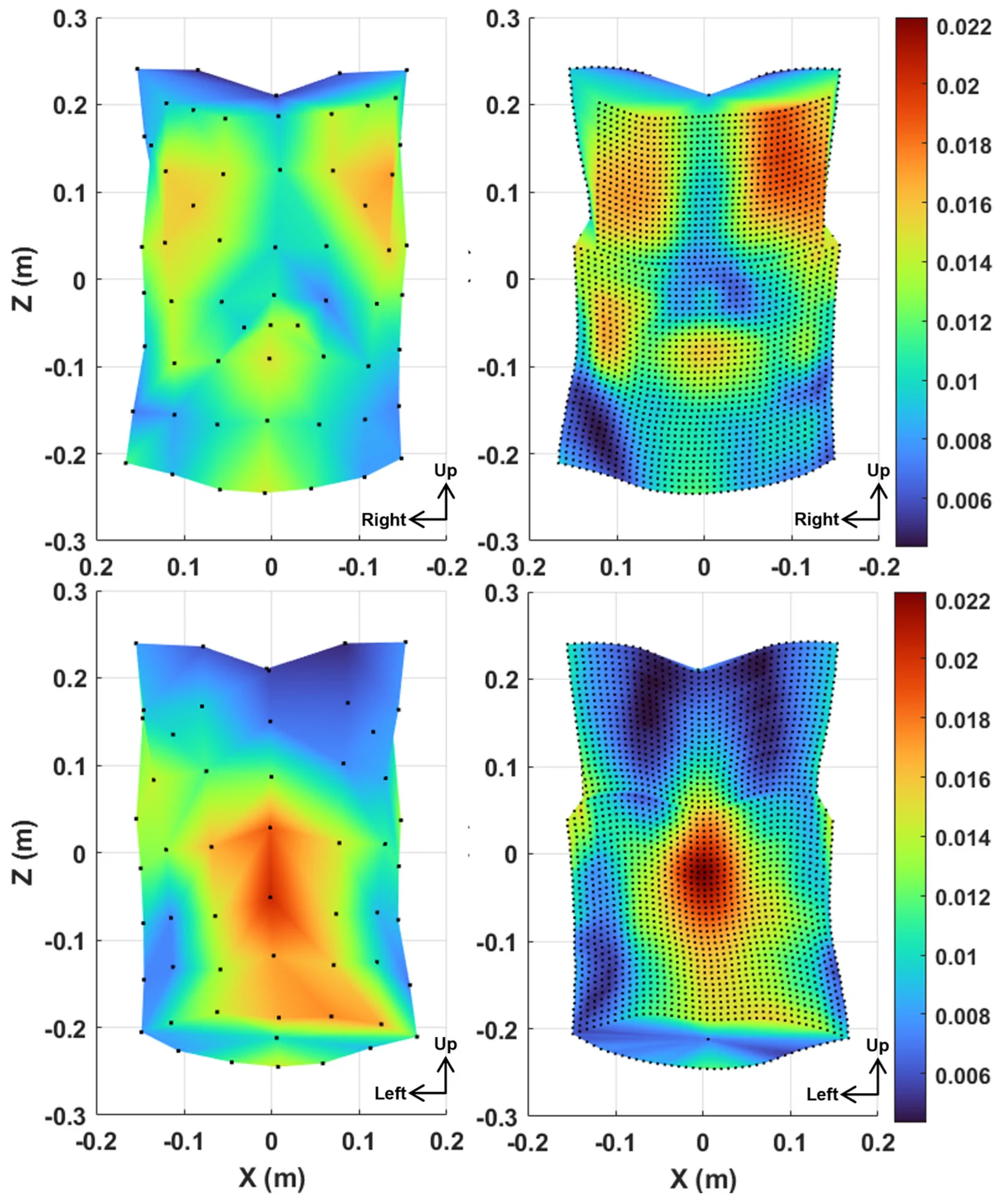
Relative radial displacements during vital capacities, represented with a colormap. (**Upper**/**lower**) panels represent anterior/posterior views respectively. (**Left**/**right**) panels represent the kinematic analysis performed with CM/ISM respectively. Colorbar indicates the amplitude of radial displacement (in m) relative to the trunk’s barycenter.

Concerning breathing motion’s kinematic analysis, an example of the potential improvement yielded by ISM is given in Figure 7. The colormap represents the radial displacement (in m) of each point delineating the chest wall surface relative to the trunk’s barycenter. The left panels represent the displacements of the original 89 markers used in CM. The right panels represent the displacements of the 4694 points generated with ISM. The anterior view with ISM (Figure 7, right upper panel) shows a higher amplitude of motion of the left upper thorax (pulmonary rib cage) compared to its right part, and a higher amplitude of motion of the right lower thorax (abdominal rib cage) compared to its left part. On the abdomen, ISM shows a lower amplitude of motion of the right iliac fossa region compared to the left one. Conversely, comparing CM and ISM posterior views (lower panels) highlights the tendency of CM to average the displacements over larger surfaces: the magnitude of the thoraco-lumbar junction motion is equivalent for both methods, but the displacement appears more diffuse with CM than with ISM.

## 4. Discussion

The aim of this study was to test ISM’s volume measurement accuracy compared to CM’s accuracy with regard to conventional spirometry. The main results of this study are as follow: (1) ISM and CM produced respiratory volume measurements similar to conventional spirometry for both small (TV) and large breathing motion (VC) in a healthy subject; (2) the linear regressions of V_OEP,ISM_ and V_OEP,CM_ with respect to V_Spiro_ for both TV and VC were equally close to the identity line, with r^2^ > 0.95; (3) the Bland–Altman analysis suggested that the ISM can be considered as accurate as CM for quiet breathing measurement; (4) for deep breathing, ISM produced a systematic bias statistically no different from zero with lower discrepancy; (5) all ICCs, for both TV and VC, were >0.968. The results of this pilot study support the interchangeability of ISM and CM for OEP volume measurement. The potential improvement of ISM for kinematic analysis has only been assessed qualitatively and requires further investigations to be considered significant.

To our knowledge, the comparison between OEP volume measurement and spirometry has been addressed in 22 studies over the past 30 years [1,2,3,4,32,33]. The overall consistency of the agreement between OEP and spirometry demonstrates the robustness of this technique, despite the wide variety in experimental conditions (standing/sitting [5,19,34], supine [7,35,36,37,38], prone [35], and lateral recumbent [39]), experimental protocols (measurements at rest [5,7,19,35,40] or before/after exercise [15,16,41,42,43]), studied population (healthy and diseased patients from adults to newborn [44,45]), and types of reference spirometer (pneumotachographs [7,15,16,37,42,43,44,45,46,47,48,49], volume displacement spirometer [7,19,35,38,40,41], turbine spirometer [21,50], hot wire spirometer [39], or ultrasonography spirometer [36]). The combined results of these studies are as follows. The range of slopes and intercepts of linear regressions are respectively [0.899; 1.21] and [−0.074; 0.201] L, with an r^2^ of [0.87; 0.99]. The overall pooled mean discrepancy is 4.5 ± 1.2%, with n = 456 subjects. The systematic bias introduced by OEP measurement was <0.068 L for healthy subjects breathing quietly. Our results for volume measurements with the CM and ISM are consistent with the existing literature for both small and large volume variations.

The agreement analyses performed in this study, including linear regression, Pearson’s correlation coefficients, Bland–Altman analysis, and intraclass correlation coefficients, provide proof of concept that the ISM algorithm can produce chest wall volume measurements comparable to those obtained with the CM algorithm during both quiet and deep breathing in a healthy subject. CM underestimated volumes in comparison with spirometry for both TV (bias = −0.015 L, *p* < 0.001, 95%CI = [−0.019; −0.011] L) and VC (bias = −0.057 L, *p* < 0.001, 95%CI = [−0.071; −0.042] L). This result is in accordance with the existing literature [5,7,19,38,41,46,48] and is also consistent with the ATS recommendation of a 3% limit for volume accuracy in spirometry [25]. For small volume variations, ISM overestimated TV with the same acceptable proportion (+0.022 L, *p* < 0.001, 95%CI = [0.016; 0.028] L). For larger volume variations (VC), ISM produced a mean bias that was not statistically different from zero (ISM = −0.001 L, *p* = 0.86, 95%CI = [−0.016; 0.013] L vs. CM = −0.057 L, *p* < 0.001, 95%CI = [−0.071; −0.042] L). The discrepancies were also lower for ISM than for CM (ISM = +0.03 ± 1.74% vs. CM = +1.91 ± 1.67%). These findings support the feasibility of the proposed approach and justify its evaluation in larger and more diverse populations.

ISM and CM differ in the following two key points: the geometrical model used and the volume computation algorithm applied.

Concerning the volume computation algorithm, two different approaches were developed at the birth of OEP. The volume approach, initiated by Ferrigno et al., leading to the PBM algorithm, involves the division of the total chest volume into a finite number of elementary tetrahedrons whose volume can be calculated from the vertices’ coordinates using the Lagrange formula [5,21]. On the other hand, the surface approach, initiated by Cala et al., leading to the CM algorithm, involves a surface integral using Gauss’ theorem, which computes the volume enclosed inside the external surface of the chest wall decomposed into a closed triangular mesh [19,35]. Although this technique has been the most widely used since the 2000s [1,3], a comparison of CM and PBM on twelve healthy subjects and 140 TV showed that PBM had the best accuracy and similarity with spirometry [34]. This explains our choice to include PBM for volume computation in ISM.

Concerning the geometrical model, both methods require supplementary virtual markers to compute the chest wall volume. ISM creates a central column composed of points, which are the centers of each ellipsoid horizontal surface delineated by the points on the chest wall [21] (Figure 1). CM needs to close the surface at the location of the shoulders, the pelvis and the neck [19] (Figure 1). The upper and lower closing points are the same for both methods. However, the closing points on the shoulders used in CM result in supplementary volumes that are absent from ISM (Figure 1). Conversely, the triangular mesh used in CM connects the points with straight lines and produces large plane facets. The modified Akima method, used twice in ISM, produces more naturally smoothed curves [35] that are anatomically more realistic (Figure 1). The absence of closing points at the shoulders and the gaps between ISM curves and CM straight lines seems to compensate each other, resulting in an OEP volume measurement with ISM as accurate as CM.

Our results suggest that differences in the geometric model do not compromise the accuracy of chest wall volume measurements, but they are likely to have a substantial impact on the kinematic analysis of breathing motion. Breathing motion alternates between inward and outward displacements of the chest wall surface. Increasing the number of points delineating the chest wall surface with ISM enhances spatial resolution, providing potential improvements in the ability to characterize local breathing kinematics. With a limited number of points, regional displacements are inherently averaged over larger surface areas, potentially masking localized motion patterns and limiting the interpretation of chest wall deformation [18]. Conversely, a denser representation of the surface provides more spatially resolved information, allowing for a more detailed characterization of the relative motion of individual regions of the chest wall. This local analysis of chest wall motion would certainly be of interest for diseased patients exerting asymmetrical motions [50]. For example, idiopathic scoliosis induces spinal and rib cage deformities without significantly affecting breathing volumes for curvatures < 40° [51]. However, OEP can identify subtle asymmetries in chest wall motion that are not reflected by conventional volume measurements [10].

A finite element method has recently been proposed to refine the OEP surface mesh by subdividing the original triangular mesh and generating a smoothed thoraco-abdominal surface [50]. After the first TurboSmooth iteration, the number of surface points increased from 93 to 548, resulting in a mean reduction in absolute chest wall volume of approximately 4.5% [50]. Although this reduction remained nearly constant across static lung volumes (from 4.5% at functional residual capacity to 4.3% at total lung capacity), the discrepancies observed for respiratory volume changes differed according to the breathing maneuver, with a median overestimation of 2.6% for tidal volume and an underestimation of 3.7% for vital capacity [50]. These findings suggest that the smoothing procedure does not behave as a simple proportional scaling of the thoraco-abdominal surface but introduces geometry-dependent modifications that vary throughout the respiratory cycle. The proposed ISM algorithm achieved a comparable level of accuracy while exhibiting more consistent behavior. Absolute chest wall volumes were overestimated by only 1.79 ± 0.22%, whereas the corresponding discrepancies for tidal volume and vital capacity were +3.63 ± 1.48% and +1.92 ± 0.49%, respectively. Although ISM cannot be considered a strictly homothetic transformation, the preservation of the bias direction and the close agreement between absolute volume and vital capacity errors suggest that the interpolation produces a more proportional geometric transformation of the thoraco-abdominal surface than TurboSmooth. This more predictable behavior may facilitate the interpretation of respiratory volume measurements while maintaining accuracy well within the limits of spirometer uncertainty.

Concerning comparison to a gold-standard measurement, five types of spirometers were used as reference in the existing literature, with a majority using pneumotachographs (PNT, 12/22 studies), followed by volume displacement (VD, 6/12 studies). Ultrasonography, turbines and hot-wire spirometers were only used once for each. One study comparing OEP, PNT and VD in four mechanically ventilated patients found discrepancies < 2% between OEP and each reference, demonstrating the feasibility and reliability of optical measurements in the intensive care unit [7]. It is noteworthy that the greatest discrepancy was observed when comparing the two reference devices (PNT vs. VD = 4.9 ± 6.4%) [7], emphasizing the limitations overcome by the optical measurements in terms of variations in air thermodynamic conditions.

The main limitation of our study concerns the sample, composed of a single healthy subject. The large number of respiratory cycles provided a robust assessment of the algorithm across repeated quiet breathing and vital capacity maneuvers for this participant. However, because all observations originated from a single individual, the findings should be interpreted as a proof of concept. The limited external validity of our results requires further investigations with a larger population and diverse morphologies to test ISM’s robustness. The two breathing conditions, quiet breathing and deep breathing, were performed without any cues concerning the frequency and the amplitude so that inherent random variability occurred. The kinematic analysis proposed in this study is exclusively descriptive and relies on a subject having no respiratory pathology. The usefulness of this improvement in detecting and quantifying abnormalities in a pathological context remains to be addressed with a conventional clinical design including a larger number of participants having various morphologies and thoracic shapes.

The risk of bias related to air leakage at the mouthpiece was minimized by the instructions to the participant and the visual control of the operator during the measurement [25]. The use of a mouthpiece is also better than the use of facemasks for avoiding leakage [48]. Nevertheless, breathing through a mouthpiece might have influenced the subject’s natural breathing pattern. The occurrence of blood shift is a common source of disagreement between OEP and spirometry [2] that we considered negligible since the flow rate for deep breathing was low.

Further studies are now required to validate the ISM’s performance in larger and more heterogeneous populations and across a broader range of thoracic morphologies and breathing patterns. Beyond volume measurements, future investigations should assess the ability of ISM to improve the characterization of local chest wall kinematics using the higher surface resolution afforded by the method and to evaluate additional respiratory outcomes derived from OEP (flows, duty cycle, inspiratory and expiratory times, etc.). Particular attention should be paid to clinical populations presenting asymmetric thoracic motion, such as individuals with scoliosis, for whom a more detailed description of regional chest wall mechanics may provide added value over conventional approaches [17].

## 5. Conclusions

Any innovation in OEP kinematic analysis has to ensure that the level of accuracy in volume measurement matches that of the previous conventional method to be considered an effective improvement. The present study provides a proof of concept for the ISM algorithm by demonstrating that increasing chest wall surface resolution can be achieved without appreciably altering the volume measurements obtained using the CM algorithm. The ISM algorithm yielded volume measurement estimates that remained highly consistent with those of CM algorithm. The trend toward improved performance for larger volumes further supports the evaluation of ISM in larger and more diverse populations encompassing a wider range of thoracic morphologies and breathing patterns. Our findings on a single participant with repeated measurements suggest that the reliability of chest wall volume measurements is not affected by the enhancement of surface resolution. As a proof of concept, this work supports the interchangeability of ISM and CM for OEP volume measurements. The added value of implementing ISM as a new tool for local chest wall kinematic analysis within the current OEP framework remains to be addressed in future studies with a larger number of participants. ISM appears to be a promising method for detailed chest wall motion analysis, providing a versatile framework that does not rely on any third-party software and an adjustable surface resolution with flexible interpolation methods.

## Figures and Tables

**Table 1:** Agreement of CM and ISM with spirometry for TV and VC measurements.

	TV		VC	
	CM	ISM	CM	ISM
Slope	0.98	1.00	0.97	1.00
Intercept (L)	0.007	0.022	0.041	0.008
Pearson r^2^	0.987	0.978	0.991	0.991
Bias (L)	−0.015	0.022	−0.057	−0.001
LOA range (L)	0.098	0.130	0.200	0.203
ICC	0.982	0.968	0.980	0.991
Discrepancy (%)	+1.44 ± 2.35	−2.15 ± 3.14	+1.91 ± 1.67	+0.03 ± 1.74

## Data Availability

The data presented in this study are available upon request from the corresponding author.

## Abbreviations

The following abbreviations are used in this manuscript:

OEP	Optoelectronic Plethysmography
CM	Conventional Method
ISM	Interpolated Surface Method
PBM	Prism-Based Method
TV	Tidal Volume
VC	Vital Capacity
B&A	Bland and Altman
ICC	Intra-class correlation

## References

[1] Massaroni C., Carraro E., Vianello A., Miccinilli S., Morrone M., Levai I.K., Schena E., Saccomandi P., Sterzi S., Dickinson J.W. (2017). Optoelectronic Plethysmography in Clinical Practice and Research: A Review. Respiration.

[2] Aliverti A., Pedotti A. (2003). Opto-electronic plethysmography. Monaldi Arch. Chest Dis..

[3] Parreira V.F., Vieira D.S.R., Myrrha M.A.C., Pessoa I.M.B.S., Lage S.M., Britto R.R. (2012). Optoelectronic plethysmography: A review of the literature. Rev. Bras. Fisioter..

[4] Romagnoli I., Lanini B., Binazzi B., Bianchi R., Coli C., Stendardi L., Gigliotti F., Scano G. (2008). Optoelectronic Plethysmography Has Improved Our Knowledge of Respiratory Physiology and Pathophysiology. Sensors.

[5] Ferrigno G., Molteni F., Carnevali P., Aliverti A. (1994). 3D kinematic analysis of respiratory movements. J. Biomech..

[6] Zannin E., Veneroni C., Dellacà R.L., Corbetta R., Suki B., Tagliabue P.E., Ventura M.L. (2018). Effect of continuous positive airway pressure on breathing variability in early preterm lung disease. Pediatr. Pulmonol..

[7] Aliverti A., Dellacá R., Pelosi P., Chiumello D., Pedotti A., Gattinoni L. (2000). Optoelectronic Plethysmography in Intensive Care Patients. Am. J. Respir. Crit. Care Med..

[8] Liou T.G., Kanner R.E. (2009). Spirometry. Clin. Rev. Allergy Immunol..

[9] West J.B. (1999). The original presentation of Boyle’s law. J. Appl. Physiol..

[10] Gaillard L., Stubbe L., Riquet D., Houel N. (2026). Chest wall motion symmetry during breathing—A systematic review with meta-analysis providing normative value in healthy subjects. Respir. Med. Res..

[11] Aliverti A., Quaranta M., Chakrabarti B., Albuquerque A.L.P., Calverley P.M. (2009). Paradoxical movement of the lower ribcage at rest and during exercise in COPD patients. Eur. Respir. J..

[12] LoMauro A., Pochintesta S., Romei M., D’Angelo M.G., Pedotti A., Turconi A.C., Aliverti A. (2012). Rib cage deformities alter respiratory muscle action and chest wall function in patients with severe osteogenesis imperfecta. PLoS ONE.

[13] Sarmento A., Fregonezi G., Dourado-Junior M.E.T., Aliverti A., de Andrade A.D., Parreira V.F., Resqueti V. (2019). Thoracoabdominal asynchrony and paradoxical motion in middle stage amyotrophic lateral sclerosis. Respir. Physiol. Neurobiol..

[14] Aliverti A., Cala S.J., Duranti R., Ferrigno G., Kenyon C.M., Pedotti A., Scano G., Sliwinski P., Macklem P.T., Yan S. (1997). Human respiratory muscle actions and control during exercise. J. Appl. Physiol..

[15] Kipp S., Leahy M.G., Hanna J.A., Sheel A.W. (2021). Partitioning the work of breathing during running and cycling using optoelectronic plethysmography. J. Appl. Physiol..

[16] Vogiatzis I., Aliverti A., Golemati S., Georgiadou O., LoMauro A., Kosmas E., Kastanakis E., Roussos C. (2005). Respiratory kinematics by optoelectronic plethysmography during exercise in men and women. Eur. J. Appl. Physiol..

[17] Gaillard L., Stubbe L., Riquet D., Houel N. (2025). Osteopathic manipulative treatment in a patient with idiopathic scoliosis assessed by breathing pattern characterization using optoelectronic plethysmography and quality of life—A case report. J. Bodyw. Mov. Ther..

[18] Solav D., Meric H., Rubin M.B., Pradon D., Lofaso F., Wolf A. (2017). Chest Wall Kinematics Using Triangular Cosserat Point Elements in Healthy and Neuromuscular Subjects. Ann. Biomed. Eng..

[19] Cala S.J., Kenyon C.M., Ferrigno G., Carnevali P., Aliverti A., Pedotti A., Macklem P.T., Rochester D.F. (1996). Chest wall and lung volume estimation by optical reflectance motion analysis. J. Appl. Physiol..

[20] Gaillard L., Stubbe L., Riquet D., Houel N. (2026). Mesh refinement of the chest wall surface to improve optoelectronic plethysmography kinematic analysis. Results Eng..

[21] Massaroni C., Cassetta E., Silvestri S. (2017). A Novel Method to Compute Breathing Volumes via Motion Capture Systems: Design and Experimental Trials. J. Appl. Biomech..

[22] Gaillard L., Stubbe L., Riquet D., Houel N. (2025). Influence of motion capture camera’s self-heating on optoelectronic plethysmography accuracy. Measurement.

[23] Boulet S., Boudot E., Houel N. (2016). Relationships between each part of the spinal curves and upright posture using Multiple stepwise linear regression analysis. J. Biomech..

[24] Stubbe L., Houel N., Cottin F. (2022). Accuracy and reliability of the optoelectronic plethysmography and the heart rate systems for measuring breathing rates compared with the spirometer. Sci. Rep..

[25] Graham B.L., Steenbruggen I., Miller M.R., Barjaktarevic I.Z., Cooper B.G., Hall G.L., Hallstrand T.S., Kaminsky D.A., McCarthy K., McCormack M.C. (2019). Standardization of Spirometry 2019 Update. An Official American Thoracic Society and European Respiratory Society Technical Statement. Am. J. Respir. Crit. Care Med..

[26] Singh S., Khan S.Z., Patel B., Gumpeni R., Verma S., Talwar A. (2021). Slow vital capacity. Int. J. Adv. Med..

[27] Cross T.J., Kelley E., Hardy T., Johnson B. (2019). The “Syringe Potentiometer”: A Low-Cost Solution for Accurate and Precise Calibration of Pneumotachographs: 2393 Board #57 May 31 9:30 AM–11:00 AM. Med. Sci. Sports Exerc..

[28] Ward M.E., Ward J.W., Macklem P.T. (1992). Analysis of human chest wall motion using a two-compartment rib cage model. J. Appl. Physiol..

[29] Kottner J., Audige L., Brorson S., Donner A., Gajewski B.J., Hróbjartsson A., Roberts C., Shoukri M., Streiner D.L. (2011). Guidelines for Reporting Reliability and Agreement Studies (GRRAS) were proposed. Int. J. Nurs. Stud..

[30] Bland J.M., Altman D.G. (2010). Statistical methods for assessing agreement between two methods of clinical measurement. Int. J. Nurs. Stud..

[31] Mcgraw K., Wong S. (1996). Forming Inferences About Some Intraclass Correlation Coefficients. Psychol. Methods.

[32] Layton A.M., Garber C.E., Basner R.C., Bartels M.N. (2011). An assessment of pulmonary function testing and ventilatory kinematics by optoelectronic plethysmography. Clin. Physiol. Funct. Imaging.

[33] Lorenzetti Branco J.H., Lorenzetti Branco R.L., Ponte Souza Filho V.P., da Silveira B., Monteiro Pause K.T., dos Santos Artismo R., Matte D.L. (2021). Can optoelectronic plethysmography be used to evaluate the thoracoabdominal kinematics of people with morbidly obesity? A systematic review. Heart Lung.

[34] Massaroni C., Senesi G., Schena E., Silvestri S. (2017). Analysis of breathing via optoelectronic systems: Comparison of four methods for computing breathing volumes and thoraco-abdominal motion pattern. Comput. Methods Biomech. Biomed. Eng..

[35] Aliverti A., Dellacà R., Pelosi P., Chiumello D., Gatihnoni L., Pedoti A. (2001). Compartmental analysis of breathing in the supine and prone positions by optoelectronic plethysmography. Ann. Biomed. Eng..

[36] Cesareo A., LoMauro A., Santi M., Biffi E., D’Angelo M.G., Aliverti A. (2018). Acute Effects of Mechanical Insufflation-Exsufflation on the Breathing Pattern in Stable Subjects with Duchenne Muscular Dystrophy. Respir. Care.

[37] Boudarham J., Pradon D., Prigent H., Falaize L., Durand M.-C., Meric H., Petitjean M., Lofaso F. (2013). Optoelectronic Plethysmography as an Alternative Method for the Diagnosis of Unilateral Diaphragmatic Weakness. Chest.

[38] Dellacà R.L., Aliverti A., Pelosi P., Carlesso E., Chiumello D., Pedotti A., Gattinoni L. (2001). Estimation of end-expiratory lung volume variations by optoelectronic plethysmography. Crit. Care Med..

[39] Nozoe M., Mase K., Takashima S., Matsushita K., Kouyama Y., Hashizume H., Kawasaki Y., Uchiyama Y., Yamamoto N., Fukuda Y. (2014). Measurements of chest wall volume variation during tidal breathing in the supine and lateral positions in healthy subjects. Respir. Physiol. Neurobiol..

[40] Gorini M., Iandelli I., Misuri G., Bertoli F., Filippelli M., Mancini M., Duranti R., Gigliotti F., Scano G. (1999). Chest Wall Hyperinflation During Acute Bronchoconstriction in Asthma. Am. J. Respir. Crit. Care Med..

[41] Kenyon C.M., Cala S.J., Yan S., Aliverti A., Scano G., Duranti R., Pedotti A., Macklem P.T. (1997). Rib cage mechanics during quiet breathing and exercise in humans. J. Appl. Physiol..

[42] Aliverti A., Stevenson N., Dellacà R.L., Lo Mauro A., Pedotti A., Calverley P.M.A. (2004). Regional chest wall volumes during exercise in chronic obstructive pulmonary disease. Thorax.

[43] Layton A.M., Moran S.L., Garber C.E., Armstrong H.F., Basner R.C., Thomashow B.M., Bartels M.N. (2013). Optoelectronic plethysmography compared to spirometry during maximal exercise. Respir. Physiol. Neurobiol..

[44] Reinaux C.M.A., Aliverti A., da Silva L.G.M., da Silva R.J., Gonçalves J.N., Noronha J.B., Filho J.E.C., de Andrade A.D., de Amorim Britto M.C. (2016). Tidal volume measurements in infants: Opto-electronic plethysmography versus pneumotachograph. Pediatr. Pulmonol..

[45] Dellaca’ R.L., Ventura M.L., Zannin E., Natile M., Pedotti A., Tagliabue P. (2010). Measurement of Total and Compartmental Lung Volume Changes in Newborns by Optoelectronic Plethysmography. Pediatr. Res..

[46] Duranti R., Filippelli M., Bianchi R., Romagnoli I., Pellegrino R., Brusasco V., Scano G. (2002). Inspiratory Capacity and Decrease in Lung Hyperinflation with Albuterol in COPD. CHEST.

[47] Vogiatzis I., Stratakos G., Athanasopoulos D., Georgiadou O., Golemati S., Koutsoukou A., Weisman I., Roussos C., Zakynthinos S. (2008). Chest wall volume regulation during exercise in COPD patients with GOLD stages II to IV. Eur. Respir. J..

[48] Barcelar Jde M., Aliverti A., Melo TLLde B., Dornelas C.S., Lima C.S.F.R., Reinaux C.M.A., de Andrade A.D. (2013). Chest wall regional volumes in obese women. Respir. Physiol. Neurobiol..

[49] Boudarham J., Pradon D., Prigent H., Vaugier I., Barbot F., Letilly N., Falaize L., Orlikowski D., Petitjean M., Lofaso F. (2013). Optoelectronic Vital Capacity Measurement for Restrictive Diseases. Respir. Care.

[50] Aliverti A., Lacca D., LoMauro A. (2022). Quantitative Analysis by 3D Graphics of Thoraco-Abdominal Surface Shape and Breathing Motion. Front. Bioeng. Biotechnol..

[51] Koumbourlis A.C. (2006). Scoliosis and the respiratory system. Paediatr. Respir. Rev..

